# Correct Assignment of Homology is Crucial When Genomics Meets Molecular Evolution

**DOI:** 10.1002/cfg.214

**Published:** 2002-12

**Authors:** Mohamed Zouine, Quentin Sculo, Bernard Labedan

**Affiliations:** 1 IGM Universit Paris-Sud Bâtiment 409 Orsay Cedex 91405 France; 2 Évolution Moléculaire et Génomique Institut de Génétique et Microbiologie CNRS UMR 8621 Université Paris-Sud Bâtiment 409 Orsay Cedex 91405 France; 3 Génomique des Microorganismes Pathogénes Institut Pasteur 25 rue du Dr Roux Paris 75015 France

## Abstract

Pertinent evolutionary studies are based on a correct use of homology terms such as paralogues, metalogues and orthologues. Such crucial concepts have been applied to intragenomic and intergenomic analyses. A further requisite is a proper definition of what is a structural segment of homology. Such segments are called modules to reflect that they play a role in the mechanism of combinational construction of a gene from ready-made basic components. Since identifying a module is operationally equivalent to determining the ancestor to this gene segment, it becomes possible to track back protein history and genome evolution. Such studies underline the importance of two fundamental processes, gene duplication and gene fusion. Moreover, grouping the closest orthologues in families is a pertinent way to reconstruct a genomic tree for all available prokaryotes.


**Comparative and Functional Genomics**
*Comp Funct Genom* 2002; **3**: 488–493.
Published online in Wiley InterScience (www.interscience.wiley.com). **DOI:** 10.1002/cfg.214

**Conference Review**

### Correct assignment of homology is crucial when genomics meets molecular evolution

Mohamed Zouine#, Quentin Sculo and Bernard Labedan*

*Évolution Moléculaire et Génomique, Institut de Génétique et Microbiologie, CNRS UMR 8621, Université Paris-Sud, Bâtiment 409, 91405 Orsay Cedex, France*

**Correspondence to: Bernard Labedan, IGM, Bâtiment 409, Université Paris-Sud, 91405 Orsay Cedex, France. E-mail: labedan@igmors.u-psud.fr*

*# Present address: Génomique des Microorganismes Pathogénes, Institut Pasteur, 25 rue du Dr Roux, 75015 Paris, France.*

Received: 9 September 2002
Accepted: 7 October 2002

### Abstract

**Pertinent evolutionary studies are based on a correct use of homology terms such as paralogues, metalogues and orthologues. Such crucial concepts have been applied to intragenomic and intergenomic analyses. A further requisite is a proper definition of what is a structural segment of homology. Such segments are called modules to reflect that they play a role in the mechanism of combinational construction of a gene from ready-made basic components. Since identifying a module is operationally equivalent to determining the ancestor to this gene segment, it becomes possible to track back protein history and genome evolution. Such studies underline the importance of two fundamental processes, gene duplication and gene fusion. Moreover, grouping the closest orthologues in families is a pertinent way to reconstruct a genomic tree for all available prokaryotes. Copyright © 2002 John Wiley & Sons, Ltd.**

**Keywords: paralogues; metalogues; orthologues; module; gene classes; ancestral genes; ancestral genomes; genomic trees**

### Introduction

Homology is one of the most important concepts in biology but its use — and misuse — has recently been extraordinarily accentuated since life scientists of nearly all fields entered the new era of genomics. Accordingly, we would remind the reader and emphasize the importance of basic, essential facts about homology (with emphasis on molecules, especially proteins). Then, to illustrate the importance of these homology concepts when using genomics data, we have summarized some of the results recently obtained using our experimental approach.

### A few definitions

### Defining homology

Two items are defined as homologues if they share a common ancestry. Such a definition has two fundamental implications: homology is (a) always a hypothesis; (b) an all-or-none property. Thus, an indirect way is necessary to assess experimentally whether two objects are homologues. In most cases, the level of similarity is the criterion used, e.g. two proteins will be labelled homologues if the number of their identical residues is higher than an imposed threshold [see e.g. 1,7].

### The different classes of homology

As early as 1970, Fitch made a fundamental distinction [9]. *Orthologous* genes are homologous genes that diverged by speciation. Therefore, orthologues are the pertinent objects to use to reconstruct phylogenetic trees. *Paralogous* genes descend from an ancestral duplication, independently of speciation. Thus, paralogues are helpful for understanding the course of protein evolution, as long as the changes to sequences over time by processes of mutation, recombination and repair have not blurred the similarities. Fitch

Copyright © 2002 John Wiley & Sons, Ltd.

also dubbed as '*xenologue*' any homologue introduced by lateral transfer. It has been further proposed [18] to use the term *metalogues* for paralogous genes that have been separated by speciation. This leads to an important point: in the case of asymmetrical loss of paralogous copies in compared species, the remaining metalogues could be erroneously interpreted as being *bona fide* orthologues.

### The minimal segment of homology

In a stochastic model of protein evolution [5,17], the evolutionary distance separating two homologous proteins is given in PAM units. A PAM unit is defined as the number of accepted point mutations per 100 residues separating two sequences. In two seminal papers [1,2] and using an approach based on information theory, Altschul, showed that 30 bits of information are necessary to distinguish an alignment from chance. He further showed that, in order to reach such a cut-off value, the length of the segment of homology was dependent on the nature of the Dayhoff substitution matrix used. Accordingly, to be statistically significant an alignment of sequences separated by a distance of 250 PAM units would need to have a length of at least 83 residues.

### The concept of module, a structural segment of homology

Riley and Labedan [16] used the Altschul cut-offs to assess the homologous relationships when comparing *Escherichia coli* proteins. In many cases, homology was found to be limited to long structural segments with a mean size of 220 amino acids. Such segments were called 'modules' to reflect that they play a role in the mechanism of combinatorial construction of a gene from ready-made basic components. In our eyes, identifying a module is operationally equivalent to determining the ancestor to this gene segment. In support of this model, it is striking that for many prokaryotic genomes, the mean size of homologous proteins ($\sim$450 residues) is about twice the size of non-homologous proteins ($\sim$250 residues), this last size being close to the module size. Our module concept is crucial to understanding protein history by taking into account two major mechanisms occurring at the gene level: duplication and fusion. As explained below, comparison of modern-day proteins and identification of all modules will help to number the events of duplication and fusion, and thus, after grouping all homologous modules in families, to trace back to the ancestral genes which were at the origin of each family.

It may be more than a coincidence that the mean size of 220 amino acids we found for prokaryotic modules is rather close to the typical fold (=structural domain) size, $150 \pm 50$, determined by a completely different approach [10,19]. Such a figure appears as a universal unit according to Wheelan *et al.* [19], supporting our model of combinatorial construction of a protein (gene) from ready-made basic components. Furthermore, we must emphasize the point that modules are conceptually entirely different from the shorter segments of homology that have been registered as domains or motifs in various specialized databases [3,4,8,14]. Domains and motifs are at a different level, being entities such as binding sites for co-factors and prosthetic groups. Domains/motifs are common features of many proteins that otherwise have no sequence similarity over a significant fraction of their total length. Although domains/motifs are important elements of the specificity of binding and the chemistry of action of a protein, as well as important features of any tertiary structure, it may be misleading to use their sequences to attempt to trace back protein history. Similar domains/motifs are found within proteins and modules that as a whole are not members of evolutionarily defined families.

### Grouping proteins in families

Families of proteins must be built only on the basis of parental relationships. Thus, clustering proteins into evolutionarily related families on the basis that they share only short domains (or worse, shared motifs) could be delusory. Another pitfall must be avoided. Some genes are multimodular in the sense that they are composed of more than one single gene now fused into one. Some fused modules have independent evolutionary histories. In forming related groups, putting in the same bag multimodular proteins having unrelated modules would be totally erroneous. For a family to be pertinent, it must be made uniquely of homologous modules [12,16].

Copyright © 2002 John Wiley & Sons, Ltd.

### Using the modular approach to study molecular evolution of genes (proteins), genomes and organisms

### Tracing back protein history

We have built a suite of automatic programs in order to cope with the present deluge of data released by the whole-genome sequencing projects [13]: (a) the Darwin AllAll program [11] detects homologous segments using thresholds for evolutionary distance (less than 250 PAM units) and alignment length (at least 80 residues); and (b) another program classifies these modules; (c) after assembling these homologous modules in families, (d) we further group families which are related by a chain of neighbouring unrelated homologous modules; (e) automatic analysis of these groups of families allows us to split into their component parts many fused modules, and/or to deduce by logic more distant modules; (f) all detected and inferred modules are reassembled in refined families. These two last steps, (e) and (f), are made by the program SortClust.

### Intragenomic analysis

Our suite identifies all kinds of modules and proteins encoded by a genome [13]. For example, in the case of *E. coli*, 5527 paralogous modules were detected. They form 1020 families, which can be separated into 307 unique families and 713 families (4586 modules) which can be clustered into 91 groups of families connected by shared modules. When applying SortClust to these 713 families, 1896 of their 4586 detected modules can be reinterpreted. The 2690 remaining detected modules and the 1295 deduced ones assemble into only 235 refined families. At the end, we get a total of 697 unique modules and 4670 modules related to at least one other module (paralogues) forming 542 families. Thus, the ancestral genome would have been made of 1607 genes ancestral to the present-day unique proteins, 542 genes ancestral to the present-day 4670 paralogous modules, and 697 genes ancestral to the components of the present-day modules (which fused in various combinations with 4670 paralogous modules to create the 2487 paralogous present-day proteins).

### Intergenomic analysis

Four classes of modules and proteins can be defined [13]. The first two categories correspond to proteins that are found in only one species (sp) and which either have a paralogue (para-sp) or are unique to their species (uni-sp). The last two categories correspond to orthologous proteins that either have a paralogue (para-ortho) or are unique to their species (uni-ortho). Figure 1 shows the respective distributions of gene classes when comparing four proteobacteria, *E. coli*, *Campylobacter jejuni, Haemophilus influenzae* and *Helicobacter pylori*. There are far more orphans, both paralogous and unique ones, in *E. coli* than in the three other bacteria. As already suggested, many of the homologues unique to *E. coli* may have been lost during the evolution of the three other proteobacteria to pathogenesis [6]. These *E. coli*-specific paralogues form a strikingly high number (411) of small families (with two to six members) coding for putative transcriptional regulators, resistance to various substances (including antibiotics), or putative membrane proteins involved in various stages of transport of metallic ions and other rare environmental substances. These different functions may be important for the survival of *E. coli* in adverse conditions.

### Tracing back genome evolution

#### Intragenomic analysis

The putative ancestral genome of *E. coli* would contain 2304 genes encoding unique modules detected or inferred in present-day proteins and 542 ancestors of the refined families of paralogous modules. Thus, a majority of genes apparently never duplicated or, if they did, all extra copies diverged very far or were eliminated. In contrast, a minority (18.5%) of genes duplicated often, with survival of the majority of the differentiated copies. Many of the products derived from the progeny of the highly duplicated minor set of ancestral genes fused either among themselves or with some of the unique genes in various combinations to increase the palette of functions available to the cell.

#### Intergenomic analysis

Thus, intragenomic data are helpful in disclosing many of the ancient events that created present-day proteins, but they are of limited use for exploring

Copyright © 2002 John Wiley & Sons, Ltd.

the distant history of genomes, since the putative size of the ancestral genome of *E. coli* would be unreasonably large. Assuming that the mean size (220 residues) of present-day modules mirrors the size of the product encoded by the 2926 'ancestral' genes, the ancestral genome would have a size as high as 1.93 Mb. However, intergenomic analysis helps one to go deeper into the past. From the data shown in Figure 1, we could tentatively reconstitute the gene distribution of the putative ancestor to the pair *E. coli*/*H. influenzae*, and that to the pair *C. jejuni*/*He. pylori*. Accordingly, the last common ancestor (LCA) to these four proteobacteria would have been made of 3207 genes: (a) 73.9% that never duplicated and have been frequently (68.5%) lost by one of the organisms after a speciation event, the rest (174) being the ancestors of the 3122 genes which form the 1301 families of orthologues found in present-day species; (b) 26.1% went through more or less frequent events of duplication, giving birth to either the 1450 members of the 600 paralogous families specific to each organism or the 7973 members of the 664 families of paralogous orthologues. The relative numbers of uni-ortho and para-ortho evolutionary units have exploded by a factor of near 18 and around 33.5, respectively (Figure 2). Such data are in strong support of the forecast hypothesis of Ohno [15] that gene duplication is the main driving force to create new proteins/functions.

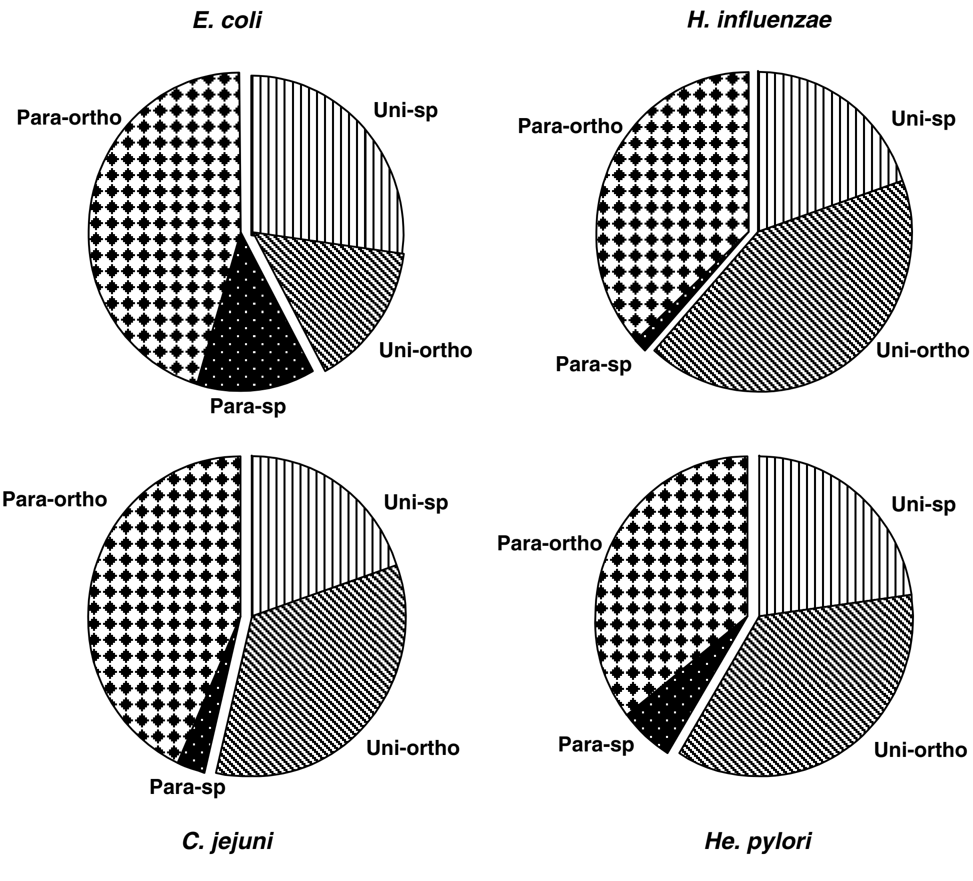

**Figure 1.** Respective proportions of gene classes in various genomes. Homology data obtained through the intergenomic comparison of the four proteobacteria *E. coli*, *H. influenzae*, *C. jejuni* and *He. pylori* are summarized in pie charts. Uni-sp, genes unique to a species without any homologue; Uni-ortho, genes unique to a species having at least one orthologue; Para-sp, paralogous genes without any homologue in another species; Para-ortho, paralogous genes having at least one orthologue

Copyright © 2002 John Wiley & Sons, Ltd. 

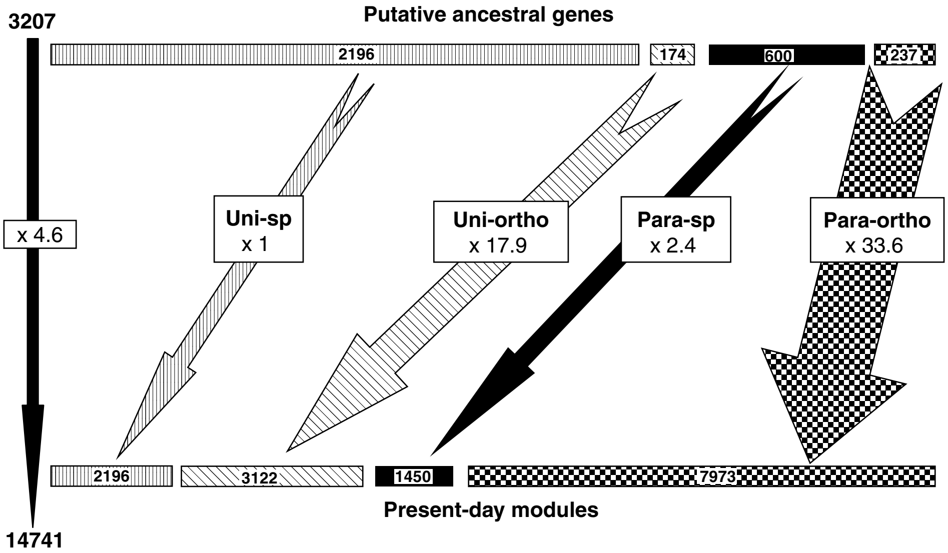

**Figure 2.** Unequal expansion of ancestral genes according to their class. See legend to Figure 1 for the definition of gene classes and symbols. The upper bars give the distribution of the four gene classes in the genome of the putative LCA (gene content, 3207), with sizes dependent on their relative percentages. The lower bars give the distribution of the contemporary modules for the same four gene classes (content in equivalent genes, 14 741) with the same size criteria. The boxed figures attached to the different arrows give the respective increase factor for each class as well as for the total (black arrow on the extreme left)

### Using families of closest orthologues to assess evolutionary relationships between prokaryotic lineages

Comparison of about 142 000 proteins present in 56 prokaryotic proteomes (46 bacteria and 10 archaea) identified more than 94 000 as having at least one orthologue. The distribution of PAM distances separating pairs of orthologues shows a nice correlation between the position of the peak of PAM distances and the phylogenetic distance separating the different pairs of organisms. On the basis of this correlation we designed the following experimental scheme: (a) selection of those orthologues having the shortest PAM distance in order to eliminate unwanted metalogues; (b) assembling these closest orthologues into 13 800 families; (c) the phylogenetic distance separating each pair of species is calculated as the mean of the means of the PAM distances separating each pair of closest orthologues computed for each family. This allows one to build a matrix of distances and to derive a tree which displays the main prokaryotic branchings found in the 16s RNA tree, but differs from it in several interesting aspects. The archaea appear to be well separated from the bacteria, with *Halobacterium* and *Thermoplasma* emerging first and in a paraphyletic position before the separation of the other euryotes (the methanogens being monophyletic), and of the crenotes. The proteobacteria are not monophyletic, the $\varepsilon$ subdivision branching far away from the node common to the $\alpha$, $\beta$ and $\gamma$ subdivisions. The chlamydiae and the spirochetes are unexpectedly positioned at the root of the bacteria, whereas the two hyperthermophiles (*Aquifex* and *Thermotoga*) form a monophyletic group that branches between the $\varepsilon$ proteobacteria and the low GC Gram-positive bacteria. *Deinococcus* groups with the high GC Gram-positive bacteria and the cyanobacteria are paraphyletic to this subgroup. *Fusobacterium* branches close to the low GC Gram-positive bacteria.

Such a global approach is interesting in that it (a) eliminates many of the flaws of the classical approach of molecular phylogeny and (b) seems more in agreement with what is known about prokaryotic biodiversity.

### Acknowledgements

We thank Monica Riley for sharing many of the concepts and ideas summarized in this article and for a critical review of this manuscript and improvement of the English. We

Copyright © 2002 John Wiley & Sons, Ltd. 

also thank the referee for valuable comments and helpful suggestions.

### References

1. Altschul SF. 1991. Amino acid substitution matrices from an information theoretic perspective. *J Mol Biol* **219**: 555–565.
2. Altschul SF. 1993. A protein alignment scoring system sensitive at all evolutionary distances *J Mol Evol* **36**: 290–300.
3. Bateman A, Birney E, Cerruti L, *et al.* 2002. The Pfam protein families database. *Nucleic Acids Res* **30**: 276–280.
4. Corpet F, Servant F, Gouzy J, Kahn D. 2000. ProDom and ProDom-CG: tools for protein domain analysis and whole genome comparisons. *Nucleic Acids Res* **28**: 267–269.
5. Dayhoff MO, Schwartz RM, Orcutt BC. 1978. A model for evolutionary change. In *Atlas of Protein Sequence and Structure*, vol 5, suppl 3, Dayhoff MO (ed.). National Biomedical Research Foundation: Washington, DC; 345–352.
6. De Rosa R, Labedan B. 1998. The evolutionary relationships between the two bacteria *Escherichia coli* and *Haemophilus influenzae* and their putative last common ancestor. *Mol Biol Evol* **15**: 17–27.
7. Doolittle RF. 1981. Similar amino acid sequences: chance or common ancestry? *Science* **214**: 149–159.
8. Falquet L, Pagni M, Bucher P, *et al.* 2002. The PROSITE database, its status in 2002. *Nucleic Acids Res* **30**: 235–238.
9. Fitch WM. 1970. Distinguishing homologous from analogous proteins. *Syst Zool* **19**: 99–113.
10. Gerstein M. 1998. How representative are the known structures of the proteins in a complete genome? A comprehensive structural census. *Folds Des* **3**: 497–512.
11. Gonnet GH, Cohen MA, Benner SA. 1992. Exhaustive matching of the entire protein sequence database. *Science* **256**: 1443–1445.
12. Labedan B, Riley M. 1999. Genetic inventory: *Escherichia coli* as a window on ancestral proteins. In *Organization of the Prokaryotic Genome*, Charlebois R (ed.). ASM Press: Washington, DC; 311–329.
13. Le Bouder-Langevin S, Capron-Montaland I, De Rosa R, Labedan B. 2002. A strategy to retrieve the whole set of protein modules in microbial proteomes. *Genome Res* (in press).
14. Letunic I, Goodstadt L, Dickens NJ, *et al.* 2002. Recent improvements to the SMART domain-based sequence annotation resource. *Nucleic Acids Res* **30**: 242–244.
15. Ohno S. 1970. *Evolution by Gene Duplication*. Springer-Verlag: New York.
16. Riley M, Labedan B. 1997. Protein evolution viewed through *Escherichia coli* protein sequences: introducing the notion of a structural segment of homology, the module. *J Mol Biol* **268**: 857–868.
17. Schwartz RM, Dayhoff MO. 1978. Matrices for detecting distant relationships. In *Atlas of Protein Sequence and Structure*, vol 5, suppl 3, Dayhoff MO (ed.). National Biomedical Research Foundation: Washington, DC; 353–358.
18. Solignac M, Periquet C, Anxolabehere D, Petit C. 1995. *Génétique et Evolution*. Hermann: Paris.
19. Wheelan SJ, Marchler-Bauer A, Bryant SH. 2000. Domain size distributions can predict domain boundaries. *Bioinformatics* **16**: 613–618.

Copyright © 2002 John Wiley & Sons, Ltd.

## References

[PDF_00399] Altschul S. F. (1993). A protein alignment scoring system sensitive at all evolutionary distances.. J Mol Evol.

[PDF_00397] Altschul S. F. (1991). Amino acid substitution matrices from an information theoretic perspective.. J Mol Biol.

[PDF_00401] Bateman Alex, Birney Ewan, Cerruti Lorenzo, Durbin Richard, Etwiller Laurence, Eddy Sean R., Griffiths-Jones Sam, Howe Kevin L., Marshall Mhairi, Sonnhammer Erik L. L. (2002). The Pfam protein families database.. Nucleic Acids Res.

[PDF_00403] Corpet F., Servant F., Gouzy J., Kahn D. (2000). ProDom and ProDom-CG: tools for protein domain analysis and whole genome comparisons.. Nucleic Acids Res.

[PDF_00415] Doolittle R. F. (1981). Similar amino acid sequences: chance or common ancestry?. Science.

[PDF_00417] Falquet Laurent, Pagni Marco, Bucher Philipp, Hulo Nicolas, Sigrist Christian J. A., Hofmann Kay, Bairoch Amos (2002). The PROSITE database, its status in 2002.. Nucleic Acids Res.

[PDF_00420] Fitch W. M. (1970). Distinguishing homologous from analogous proteins.. Syst Zool.

[PDF_00422] Gerstein M. (1998). How representative are the known structures of the proteins in a complete genome? A comprehensive structural census.. Fold Des.

[PDF_00425] Gonnet G. H., Cohen M. A., Benner S. A. (1992). Exhaustive matching of the entire protein sequence database.. Science.

[PDF_00436] Letunic Ivica, Goodstadt Leo, Dickens Nicholas J., Doerks Tobias, Schultz Joerg, Mott Richard, Ciccarelli Francesca, Copley Richard R., Ponting Chris P., Bork Peer (2002). Recent improvements to the SMART domain-based sequence annotation resource.. Nucleic Acids Res.

[PDF_00441] Riley M., Labedan B. (1997). Protein evolution viewed through Escherichia coli protein sequences: introducing the notion of a structural segment of homology, the module.. J Mol Biol.

[PDF_00451] Wheelan S. J., Marchler-Bauer A., Bryant S. H. (2000). Domain size distributions can predict domain boundaries.. Bioinformatics.

[PDF_00411] de Rosa R., Labedan B. (1998). The evolutionary relationships between the two bacteria Escherichia coli and Haemophilus influenzae and their putative last common ancestor.. Mol Biol Evol.

